# Chest wall syndrome among primary care patients: a cohort study

**DOI:** 10.1186/1471-2296-8-51

**Published:** 2007-09-12

**Authors:** François Verdon, Bernard Burnand, Lilli Herzig, Michel Junod, Alain Pécoud, Bernard Favrat

**Affiliations:** 1 Institute of General Medicine, University of Lausanne, Lausanne, Switzerland; 2 Clinical Epidemiology Centre, Institute of Social and Preventive Medicine, University of Lausanne, Lausanne, Switzerland; 3 Department of Ambulatory Care and Community Medicine, University of Lausanne, Lausanne, Switzerland

## Abstract

**Background:**

The epidemiology of chest pain differs strongly between outpatient and emergency settings. In general practice, the most frequent cause is the chest wall pain. However, there is a lack of information about the characteristics of this syndrome. The aims of the study are to describe the clinical aspects of chest wall syndrome (CWS).

**Methods:**

Prospective, observational, cohort study of patients attending 58 private practices over a five-week period from March to May 2001 with undifferentiated chest pain. During a one-year follow-up, questionnaires including detailed history and physical exam, were filled out at initial consultation, 3 and 12 months. The outcomes were: clinical characteristics associated with the CWS diagnosis and clinical evolution of the syndrome.

**Results:**

Among 24 620 consultations, we observed 672 cases of chest pain and 300 (44.6%) patients had a diagnosis of chest wall syndrome. It affected all ages with a sex ratio of 1:1. History and sensibility to palpation were the keys for diagnosis. Pain was generally moderate, well localised, continuous or intermittent over a number of hours to days or weeks, and amplified by position or movement. The pain however, may be acute. Eighty-eight patients were affected at several painful sites, and 210 patients at a single site, most frequently in the midline or a left-sided site. Pain was a cause of anxiety and cardiac concern, especially when acute. CWS coexisted with coronary disease in 19 and neoplasm in 6. Outcome at one year was favourable even though CWS recurred in half of patients.

**Conclusion:**

CWS is common and benign, but leads to anxiety and recurred frequently. Because the majority of chest wall pain is left-sided, the possibility of coexistence with coronary disease needs careful consideration.

## Background

Chest pain is a frequent complaint in ambulatory care, and while well described in emergency settings, it is less well studied in general practice. The causes are diverse, including a broad spectrum from life threatening diseases to benign causes such as chest wall syndrome (CWS) [1,2]. This painful condition of the anterior chest wall is caused by a musculoskeletal disorder and associated with tenderness of the chest wall. However, the benignity of this syndrome should be questioned when considering that it can produce for patients, greater impairment in daily activities, emotional distress and a higher level of anxiety than ischemic heart disease [3]. Furthermore, despite reassurances, a substantial proportion of patients think that they have cardiac disease [4]. Despite the high prevalence of this syndrome often seen by the general practitioners (GP) [5], prospective studies in general practice are lacking. Incidence, clinical manifestations and evolution are poorly defined. Although potentially considered as one entity -the chest wall syndrome- clinicians often prefer individual terminology depending on points of maximum tenderness on the chest wall. Historically, these different syndromes have been described separately but have not been described together [6-17]. Furthermore, in practice, multiple names have been used to describe this syndrome such as costochondritis, anterior chest wall syndrome, atypical chest pain, musculoskeletal chest pain syndromes... probably reflecting the poor understanding of this condition [9]. The aim of this prospective study was to describe the clinical characteristics and classification of CWS with long term follow-up by 300 consecutive cases seen in ambulatory practices diagnosed by GPs as CWS.

## Methods

58 GP's in private practices included all consecutive patients presenting with thoracic pain (as they main or ancillary symptom) over a 3 to 9 (median 5)-week period from March to May 2001.

Physicians recorded their observation and their diagnostic hypotheses on questionnaires developed for the study by the research group which include GPs and specialists in clinical epidemiology. The questionnaires were validated in a pilot study but were not used elsewhere. All questionnaires were filled out immediately after identifying a complaint of chest pain and after each step of consultation: the initial appraisal, completed history, physical exanimation, emergency examinations and at the end of the index encounter. The suspected diagnosis was noted after each step, as were detailed history and physical examination, level of anxiety expressed by patients and physicians, cardiovascular and thrombo-embolic risk factors, laboratory results in emergency, comorbitidies, medication and treatment decision at the end of the consultation. The questionnaire included 58 items for history including precise description of pain, provoking factors, duration, evolution, intensity, quality, modification with position, ancillary symptoms and open text to describe the chest pain as well as precise localisation on an anatomical map. Physical signs included 22 items in 5 anatomical systems: general signs, cardiovascular, respiratory, parietal, neurological, and psychiatric signs.

Questionnaires at 3 months and 12 months evaluated new complaints, investigations, new treatments, hospitalisations, and deaths. For the follow-up, we performed ambulatory check-up examinations or a telephone interview when necessary (5% patients).

The specific diagnosis retained by GPs at the end of the initial encounter was compared with the 3 and 12-month diagnosis. The final diagnoses were reviewed independently by a group of clinicians and discussed in the case of inconsistency. In the case of a new diagnosis, the GPs were contacted to confirm or reject the alternative diagnosis. The GPs sent all filled out questionnaires to the study coordination centre. All questionnaires were doubled entered by an independent company to ensure accuracy. For analysis, we presented the 300 cases with chest wall syndrome. We preferred to retain the 3-month diagnosis as the follow-up was perfect (100%).

In the data analysis, the *t *test was used for continuous variables, while the chi-square test was employed for data expressed as proportions. All statistical analyses were performed with Statview 5.0 or Stata 7.0. Clinical factors significantly associated with having a CWS were identified in an univariate analysis. To precisely determine independent indicators of a CWS, factors identified in univariate analysis were introduced in a multivariate logistic regression.

This study was approved by the local ethics committee of the Medical Faculty (University of Lausanne) and each patient gave consent to study participation.

## Results

### Patients

Among 24 620 primary care encounters, 672 consecutive patients presenting with thoracic pain (2.7%) were included. At 3-month follow-up, the main diagnostic groups were: thoracic wall pain 51% (CWS 44.6%, traumatic 4%, divers 2.4%), cardiovascular diseases 16% (coronary artery diseases CAD 12.5%, non CAD 3.4%), psychogenic pain 11%, respiratory diseases 10%, gastrointestinal disorders 8%, and no diagnosis 4%. CWS (300 cases), represented 1.2% of all consultations, and was thus more common than coronary artery diseases (CAD) (84 cases or 0.34%). Follow-up amounted to 100% and 97% at 3 and 12 months, respectively.

For the CWS, we found 155 women and 145 men. The overall mean age was 50.3 ± 18.2. The CWS was found for all range of ages including teenagers and patients more than 75. CWS was new in 62% of cases, old or recurrent in 35% and indeterminate in 3%. In half of the patients, the complaint was the principal one. 90% of the patients were already known by the GPs meaning the patient was occasionally or regularly under the care of the GPs. Comorbidity was noted for 250 patients (83%) with most notably for psychiatry comorbidity (149 patients; 50%), cardiovascular diseases (100 patients, 33.3% among whom 19 coronary diseases, 6%) or rheumatologic disease (62 patients, 20.7%, including 2 cases of spondyloarthritis but no rheumatoid diseases). Six patients had neoplasic diseases, including two lung cancers.

#### Clinical characteristics

The pain intensity was described as moderate by 75% of patients, and severe by 23%, and lasts hours, days or weeks. In a majority of patients (71%), it is amplified by factors such as a specific position or motion, by lying position, and breathing. The patients often reports bouts of pain lasting from a few seconds to days (50%) or continuous pain for hours to weeks (35%). However, acute and intense pain can happen (9%). The reported pain is mostly well-localized. The left side is predominantly affected compared to the right side. Pain can radiate to the left arm (18 patients) or the right arm (4), the back (5), the neck or the abdomen (3 patients respectively). Palpation of the thorax often reveals a tender point within the reported painful area. This is present in 214 of the 300 patients. The GPs reported a positive response to provocative manoeuvres in some patients but these haven't been systematically identified.

CWS can be associated with original characteristics such as coughing or long lasting dyspnoea, pre-existing anxious state, thoracotomy, or various rheumatologic conditions (table 1). In 8 patients, GPs diagnose a "costovertebral disorder" related to an unilateral anterior pain together with a spine disorder. No tender point is recorded as the pain probably irradiates from the spine. Short repeated and localized stabs of pain corresponding to the precordial catch [18] might even be experienced (3 patients). This kind of pain is frequent but rarely the cause of a consultation. "Thoracic catch" may be a preferable term because it can be experienced on the right side of the chest too.

**Table 1:** Influence of associated conditions on the clinical manifestation of CWS

**Associated conditions**	**n *(%)***	Characteristics versus the other cases of CWS *
Long lasting cough or dyspnea	31 *(10)*	Mostly a recent complaint More frequently right-sided pain (26 vs 11%)
Anxiety	20 *(7)*	Lower mean age (43 vs 51 year) Constrictive pain (55 vs 23%) Initiated by an anxious mood (40 vs 11%)
Fibromyalgia	10 *(3)*	9 of 10 being female Wide spread pain; frequent irradiation
After thoracotomy	8 *(3)*	Long duration and recurrent CWS
Rheumatic disorder	8 *(3)*	Cyphosis, osteoporosis (3), spondylarthritis (2), arthritis (2), radiotherapy (1)
Spine disorder	8 *(3)*	Unilateral pain without tender point, spine disorder

* all differences significative at p < 0.05

The CWS frequently causes anxiety for patients and for doctors. 54% of patients experiencing moderate intensity pain expressed anxiety compared with 93% of the patients experiencing acute and intense pain. In the first case, 10% of the doctors expressed concern or think of serious trouble, and 26% in the second case.

#### Distinctive characteristics

Compared to other non-rheumatic thoracic pain, CWS has few distinctive characteristics but the clustering of predictors led to a more precise picture. The pattern of the six most determinants predictors allowed the attribution of a clinical score to the patient (one point for each one of the six factors present) (table 2). The presence of four factors or more predict the CWS with a sensibility of 0.82 (IC95% 0.78–0.86) and a specificity of 0.54 (0.48–0.59) and for five or six factors values are 0.61 (0.55–0.66) and 0.83 (0.79–0.87).

**Table 2:** Clinical characteristics of Chest Wall Syndrome (CWS) versus the other conditions causing chest pain

**The six most discriminative clinical characteristics of CWS**	**Logistic regression Odd Ratio (IC95%)**^1^
Pain is	
- not squeezing nor oppressive	2.53 (1.21–5.28)
- localised on the left or median-left part of the chest wall	2.28 (1.58–3.28)
- well localised on the chest wall	2.10 (1.37–3.22)
- non exercise-induced chest pain	1.58 (1.00–2.49)
- influenced by mechanical factors^2^	1.54 (1.06–2.24)
- reproducible by palpation	5.72 (1.20–5.28)

^1 ^Clinical factors significantly associated with having a CWS were identified in univariate analysis then introduced in a multiple logistic regression

^2^A movement or a body position

#### The individual syndromes of CWS

**Figure 1:**
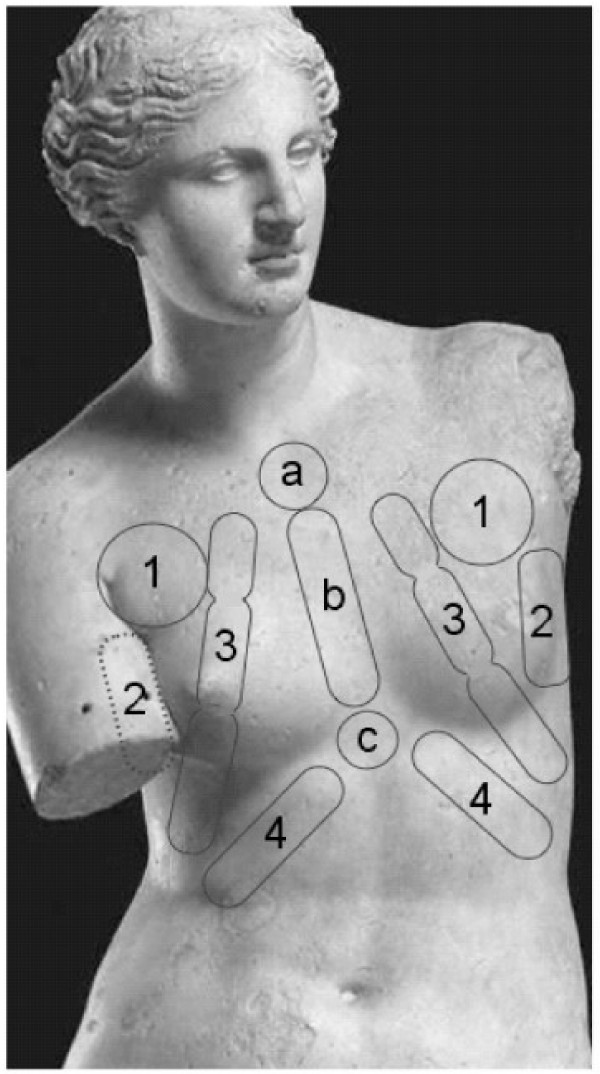
Localisation of points of maximum tenderness in the chest wall. Letters and numbers refer to the individual syndromes listed in Table 3.

Francophone patients familiarly speak of"intercostal rheumatism". GPs differ in their use of vocabulary such as "musculoskeletal pain, parietal or intercostal pain, Tietze's syndrome, chondrocostal pain, slipping rib syndrome, etc". CWS could be considered as an entity but may often be characterised as individual syndromes defined by different points of maximum tenderness on the chest wall. These different syndromes are described individually in studies and more systematically in some textbooks [19-21]. In this study, a individual syndrome can be defined in 195 of the 210 patients suffering from localized pain. The thoracic pain of 88 patients spread widely whereas for 17 patients localisation was too diffuse to give a precise definition. The most frequent individual syndromes are the left chondrocostal syndrome (70 patients), the left pectoral syndrome (40 patients), and the sternal syndrome (28) (Table 3 and Figure 1). The most frequent association of symptoms is the sternal with the chondrocostal syndrome. Some patients (26) complain of chest pain on both sides of the thorax. We have added to the commonly described syndromes the axillary (or laterothoracic) syndrome, which is quite frequent.

**Table 3:** The different syndromes of the chest wall syndrome

**Thoracic sites**^1 ^**and the 195 individual CWS *(%)***								
**Individual CWS**	**right**		**middle**		**Left**		**Synonyms and references**	

Upper sternalis s.			**a**	*(2.6)*				
Sternalis s.			**b**	*(14.4)*				^8,17^
Xiphoidalgia			**c**	*(3.1)*			xiphoidal, xiphodynia	^6,8,11^
Pectoralis s.	**1**	*(3.1)*			**1**	*(20.5)*	algia pectoralis	^15,17^
Axillary s.	**2**	*(2.6)*			**2**	*(6.2)*	laterothoracic s.	
Chondrocostal s.	**3**	*(6.2)*			**3**	*(35.8)*	costochondral, costochondritis, sternocostal or costosternal or Tietze's. if swelling	^8,9,14^
upper (C 2–3)		*(1.0)*				*(8.7)*		
mid (C 4–6)		*(2.1)*				*(19.0)*		
lower (C 7–9)		*(3.1)*				*(8.2)*		
Rib Tip s.	**4**	*(2.6)*			**4**	*(3.1)*	Lower rib, slipping rib, slipping cartilage, clicking rib, Cyriax's	^10,12^

*total*		*(14.5)*		*(20.0)*		*(65.5)*		

Numbers indicate the percentage of the 195 patients with an isolated syndrome. The most frequent syndromes are chondrocostal syndromes, particulary the mid chondrocostal, pectoralis and sternalis syndromes. Note the prevalence of left-sided syndromes

^1 ^Sites are shown on Figure 1

#### Evolution

During the year of follow-up, 171 patients (57%) were found to have suffered CWS more than once. Four patients died and 6 were hospitalised for causes independent of CWS. One patient was hospitalised and diagnosed with CWS. One patient was newly diagnosed with myocardial infarction and 3 with coronary heart disease; the initial diagnosis of CWS was not questioned in any of these cases. On the other hand, the initial diagnosis of CWS was abandoned in 16 patients for whom oesophagitis (7), anxiety or somatizing (4), coronary heart disease (3), lung cancer (1) and parietal metastases (1) were diagnosed. This illustrates the variety of conditions with which CWS can be confused.

## Discussion

In this prospective study, CWS was present in 1.2% of the consultations. Thus, CWS was responsible for 44.6% of chest pain cases, for which it was by far the most frequent cause. Indeed, it was three times more frequent than pains of cardiac origin. On average, GPs encountered one to two cases a week. This prevalence agrees with other studies in ambulatory settings [5,22,23]. Built exclusively on history and physical attributes, the diagnosis is a real challenge [24-26]. The presence of one or several points sensitive to gentle fingerprint palpation is an important sign, even if GPs sometimes diagnose CWS without it. Nevertheless, tender points are only of diagnostic significance if they match the localization of spontaneous pain, which is mostly unilateral. The presence of a tender point is far from specific for the diagnosis of CWS, as it is frequently recorded in affections of other origins such as coronary heart disease [6,27], pulmonary embolism [28], pleuritic, neoplasic and psychogenic diseases. However, our community-based study goes against existing information, namely, that half of patients with angina pectoris have tender points [6], and that half patients with chest wall tenderness suffer from coronary heart disease [27]. The same consideration is probably valid in the case of the manoeuvres of provocation, even though their sensitivity and their specificity is also unknown [19].

The strong prevalence of pain on the left side of the chest is intriguing. It is mentioned only by Lipkin et al to our knowledge [13] but is apparent in an emergency department [29]. Only speculative hypotheses could be proposed as innervation is predominantly left sided because of the presence of the heart. Therefore the sensitive efferences converge to the same central neurones than do those from the chest wall.

The aetiology of CWS is usually ill-defined. However, the painful areas are found in zones of muscular or tendinous insertions on the bones, or on cartilage or mobile zones of bone-cartilage transitions, such as the costochondral junctions. Actual inflammatory lesions are probably an exception as suggested by the absence in our study of cases of real Tietze' syndrome and of rheumatoid arthritis, a well-known inflammatory soft tissue disorder. Factors favouring pain may not be the same in all patients as disorders such as traumatic origins following a long lasting cough, thoracotomy, or an overuse of respiratory muscles in the case of asthma can all give rise to CWS. In situations of anxiety and tension, Bass et al suggest a mechanism of amplification of the pain in which anxiety increases the common sensibility of a specific point that the patient believes is a threatening somatic condition, thus increasing anxiety [30,31].

To give a precise diagnosis of CWS is important for the patients suffering from chest pain and fearing a life threatening disease. A clear diagnosis of CWS is reassuring in comparison of just ruling out a serious disease without a precise diagnosis. Furthermore, the definition of a precise syndrome is more than a mere academic interest. This makes understandable the particularities attached to certain chest wall syndromes. For instance, the pectoral syndrome (and even the axillary syndromes) is the cause of practically all acute presentations of CWS, and patients commonly suspect these syndromes to be coronary disease or, in the case of women, to be breast cancer. The muscular origin can explain the frequent radiation of pain to the left arm [32]. The upper sternalis syndrome can have an usual musculoskeletal origin but may be the result of pathologies of the sternoclavicular joint such as subluxation, arthritis, SAPHO syndrome or of pathologies of the manubriosternal joint associated with psoriasis and spondylarthropathies, as in the case of two of our patients [33,34]. As for xiphoidal and lower rib syndromes, they can last a very long time, sometimes decades, and be mistaken for digestive diseases to such a degree that publications concerning this problem are issued by gastroenterology services [35,36].

There are caveat to our data. It is possible that a benign affection such as CWS has been underreported by GPs. It is not clear that all the CWS characteristics were systematically reported in all the patients, and that even all important questions were asked in the questionnaire. Moreover, standard criteria for the diagnosis were lacking and the diagnosis was generally made without outside supervision. In this survey, some diagnostic errors are inevitable. However, the follow-up after one year may clarify cases in which severe conditions were mistaken for CWS. On the other hand, it is also possible that the year of follow-up, together with the CWS-motivated investigation, allowed accompanying conditions to be identified. These may include cases of coronary heart disease that GPs misdiagnosed as simple CWS. Another confusing factor lies in the fact that some chest wall pain could have its origin outside the anterior chest wall if the precordial catches and "vertebrothoracic" disorders are taken into consideration [37,38]. Nevertheless, we think that these ambiguous cases are few, and that our patients' samples are sufficient to describe CWS accurately. CAD and CWS may coexist as it is evident after a thoracotomy for a CAD. A study made in a academic emergency room during the same period and covering the same region as our study shows three major differences: CAD is the major cause of chest pain in the emergency room, acute coronary syndrome is quite common, and CWS cases are mostly described as acute. This suggests that this rare presentation of CWS drives patients to seek the care of emergency centres [39].

## Conclusion

CWS is a frequent condition with good prognosis, low morbidity and no mortality. Evolution has only been negative in cases of misdiagnosis, mainly in the presence of malignant conditions or in cases of coincidental diseases such as heart or neoplasic disease or pneumonitis. Nevertheless, this condition tends to recur. It also causes real anxiety and frequently suggests to the patient the possibility of heart disease. Moreover, in a few cases, it is difficult to distinguish between coronary heart disease and CWS on a clinical basis partly because the majority of chest wall pain is left-sided.

## Competing interests

The authors declare that they have no competing interests.

## Authors' contributions

FV, BB, LH, and BF participated in the conception and design of the study, analysis and interpretation of data, drafting and revising the manuscript, and inclusion of patients for BF, FV and LH. AP participated in the conception and design of the study, drafting and revising the manuscript. BF will act as guarantor for the paper.

## Pre-publication history

The pre-publication history for this paper can be accessed here:



## References

[B1] Richter JE (1991). Practical approach to the diagnosis of unexplained chest pain. Med Clin of N Amer.

[B2] Eslik GD, Coulshed DS, Talley NJ (2002). Review article: the burden of illness of non-cardiac chest pain. Aliment Pharmacol Ther.

[B3] Lau GK, Hui WM, Lam SK (1996). Life events and daily hassles in patients with atypical chest pain. Am J Gastroenterol.

[B4] Wielgosz AT, Fletcher RH, McCants CB, McKinnis RA, Haney TL, Williams RB (1984). Unimproved chest pain in patients with minimal or no coronary disease: a behavioral phenomenon. Am Heart J.

[B5] Svavarsdottir AE, Jonasson MR, Gudmundsson GH, Fjeldsted K (1996). Chest pain in family practice. Diagnosis and long-term outcome in a community setting. Can Fam Physician.

[B6] Prinzmetal M, Massumi RA (1955). The anterior chest wall syndrome: chest pain resembling pain of cardiac origin. J Amer med Ass.

[B7] Wehrmacher WH (1958). The painful anterior chest wall syndromes. Med Clin North Am.

[B8] Epstein SE, Gerber LH, Borer JS (1979). Chest wall syndrome. A common cause of unexplained cardiac pain. JAMA.

[B9] Calabro JJ, Jeghers H, Miller KA, Gordon RD (1980). Classification of anterior chest wall syndromes. JAMA.

[B10] Tietze A (1921). Über eine eigenartige Haufund von Fallen mit Dystrophie der Rippenknorpel. Berlin Klin Wschr.

[B11] Wolf E, Stern S (1976). Costosternal syndrome: its frequency and importance in differential diagnosis of coronary heart diseases. Arch Intern Med.

[B12] Davies-Colley R (1922). Slipping rib. BMJ.

[B13] Lipkin M, Fulton LA, Wolfson EA (1955). The syndrome of the hypersensitive xiphoid. N Engl J Med.

[B14] Heinz GJ, Zavala DC (1977). Slipping rib syndrome. JAMA.

[B15] Fam AG (1988). Approach to musculoskeletal chest wall pain. Prim Care.

[B16] Wise CM, Semble EL, Dalton CB (1992). Musculoskeletal chest wall syndromes in patients with noncardiac chest pain: a study of 100 patients. Arch Phys Med Rehabil.

[B17] Verdon F, Studer JP (1988). Left-sided pectobrachialgia. Schweiz Med Wochenschr.

[B18] Miller AJ, Texidor TA (1959). The "precordial catch", a syndrome of anterior chest pain. Ann Int Med.

[B19] Reilly BD (1991). Pratical strategies in outpatient medecine.

[B20] UpToDate 34 Washington Street Suite 100 Wellesley, MA 02481-1903.

[B21] Sheon RP, Moskowitz RW, Goldberg VM (1996). Thoracic and chest wall disorders. Soft Tissue Rheumatic Pain Recognition, Management, Prevention.

[B22] Buntinx F, Truyen J, Embrechts P, Moreel G, Peeters R (1991). Chest pain: an evaluation of the initial diagnosis made by 25 Flemish general practitioners. Fam Pract.

[B23] Klinkman MS, Stevens D, Gorenflo D (1994). Episodes of care for chest pain: a preliminary report from MIRNET. Michigan Research Network. J Fam Pract.

[B24] Constant J (1983). The clinical diagnosis of nonanginal chest pain: the differentiation of angina from nonanginal pain by history. Clin Cardiol.

[B25] Goodacre S, Locker T, Morris F, Campbell S (2002). How useful are clinical features in the diagnosis of acute, undifferentiated chest pain. Acad Emerg Med.

[B26] Cooke RA, Smecton N, Chambers JB (1997). Comparative study of chest pain characteristics in patients with normal and abnormal coronary angiograms. Heart.

[B27] Cohen BR (1970). Evaluation of the patient with chest pain. Modern Treatment (New York).

[B28] Le Gal G, Testuz A, Righini M, Bounameaux H, Perrier A (2005). Reproduction of chest pain by palpation: diagnostic accuracy in suspected pulmonary embolism. BMJ.

[B29] Eslick GD (2005). Usefulness of chest pain character and location as diagnostic indicators of an acute coronary syndrome. American Journal of Cardiology.

[B30] Bass C, Wade C (1984). Chest pain with normal coronary arteries: a comparative study of psychiatric and social morbidity. Psychol Med.

[B31] Mayou R (1998). Chest pain, palpitations and panic. J Psychosom Res.

[B32] Kellgren JH (1939). On distribution of pain arising from deep somatic structures with charts of segmental pain areas. Clin Sci.

[B33] Kahn MF, Chamot AM (1992). SAPHO syndrome. Rheum Dis Clin North Am.

[B34] Dawes PT, Sheeran TP, Hothersall TE (1988). Chest pain–a common feature of ankylosing spondylitis. Postgrad Med J.

[B35] Scott EM, Scott BB (1993). Painful rib syndrome–a review of 76 cases. Gut.

[B36] Baldi F, Ferrarini F (1995). Non-cardiac chest pain: a real clinical problem. Eur J Gastroenterol Hepatol.

[B37] Brown CW, Deffer PA, Akmakjian J, Donaldson DH, Brugman JL (1992). The natural history of thoracic disc herniation. Spine.

[B38] Arroyo JF, Jolliet P, Junod AF (1992). Costovertebral joint dysfunction: another misdiagnosed cause of atypical chest pain. Postgrad Med J.

[B39] Wasserfallen JB, Berger A, Eckert P, Stauffer JC, Schlaepfer J, Gillis D, Cornuz J, Schaller MD, Kappenberger L, Yersin B (2004). Impact of medical practice guidelines on the assessment of patients with acute coronary syndrome without persistent ST segment elevation. Int J Qual Health Care.

